# Seasonality and interannual variability of CH_4_ fluxes from the eastern Amazon Basin inferred from atmospheric mole fraction profiles

**DOI:** 10.1002/2015JD023874

**Published:** 2016-01-14

**Authors:** Luana S. Basso, Luciana V. Gatti, Manuel Gloor, John B. Miller, Lucas G. Domingues, Caio S. C. Correia, Viviane F. Borges

**Affiliations:** ^1^ Atmospheric Chemistry Laboratory, Instituto de Pesquisas Energéticas e NuclearesComissão Nacional de Energia NuclearSão PauloBrazil; ^2^ School of GeographyUniversity of LeedsLeedsUK; ^3^ Global Monitoring Division, Earth System Research LaboratoryNational Oceanic and Atmospheric AdministrationBoulderColoradoUSA

**Keywords:** methane, Amazon, greenhouse gas, methane flux

## Abstract

The Amazon Basin is an important region for global CH_4_ emissions. It hosts the largest area of humid tropical forests, and around 20% of this area is seasonally flooded. In a warming climate it is possible that CH_4_ emissions from the Amazon will increase both as a result of increased temperatures and precipitation. To examine if there are indications of first signs of such changes we present here a 13 year (2000–2013) record of regularly measured vertical CH_4_ mole fraction profiles above the eastern Brazilian Amazon, sensitive to fluxes from the region upwind of Santarém (SAN), between SAN and the Atlantic coast. Using a simple mass balance approach, we find substantial CH_4_ emissions with an annual average flux of 52.8 ± 6.8 mg CH_4_ m^−2^ d^−1^ over an area of approximately 1 × 10^6^ km^2^. Fluxes are highest in two periods of the year: in the beginning of the wet season and during the dry season. Using a CO:CH_4_ emission factor estimated from the profile data, we estimated a contribution of biomass burning of around 15% to the total flux in the dry season, indicating that biogenic emissions dominate the CH_4_ flux. This 13 year record shows that CH_4_ emissions upwind of SAN varied over the years, with highest emissions in 2008 (around 25% higher than in 2007), mainly during the wet season, representing 19% of the observed global increase in this year.

## Key Points

- Long time series shows substantial CH_4_ emissions in eastern Brazilian Amazon
- Higher CH_4_ emissions in the beginning of wet season and during the dry season
- Higher 2008 emissions recorded at SAN contribute to global increase of CH_4_

## 1 Introduction

CH_4_ is the second most important anthropogenic greenhouse gas after CO_2_, contributing approximately 18%, or 0.48 Wm^−2^ to present anthropogenic greenhouse warming [*Intergovernamental Panel on Climate Change (IPCC),* 2013; *World Meteorological Organization, Global Atmosphere Watch, World Data Centre for Greenhouse Gases,* 2014]. Although the atmospheric mole fraction of CH_4_ is approximately 200 times lower than that of CO_2_, its global‐warming potential is approximately 28 times higher than CO_2_ when calculated over a 100 year period [*IPCC,* 2013]. The levels of CH_4_ in the atmosphere are lower than the CO_2_ levels primarily because CH_4_ undergoes oxidation in the atmosphere, particularly with OH, leading to an atmospheric lifetime for CH_4_ of around 9 years [*Prather et al.,* 2012]. Since 1750, the global atmospheric CH_4_ mole fraction has been increasing from around 700 ppb in 1750 [*Etheridge et al.,* 1998] to around 1800 ppb in 2012 [*WMO, GAW, WDCGG,* 2014].

From 1999 to 2006 the global atmospheric CH_4_ growth rate unexpectedly stalled, indicating that the emissions were equal to CH_4_ destruction [*Dlugokencky et al.,* 2003]. However, after 2007, atmospheric measurements have shown renewed global atmospheric CH_4_ growth [*Rigby et al.,* 2008; *Dlugokencky et al.,* 2009; *Nisbet et al.,* 2014]. The drivers of this renewed growth are still being debated, and the reasons remain incompletely understood [*Nisbet et al.,* 2014]. The increase in the global atmospheric CH_4_ mole fraction was around 2 ppb during the period from 2000 to 2006 (equivalent to ~5 Tg yr^−1^, if sinks were constant) and around 32 ppb between 2007 and 2013 (equivalent to ~89 Tg), based on regularly performed measurements at the NOAA/GMD Global Greenhouse Gas Reference Network which covers the globe [*Dlugokencky et al.,* 2015]. Two main factors have been named as likely explanations for this recent renewed increase. First, very warm temperatures at high northern latitudes during 2007 likely enhanced emissions from northern wetlands. Second, positive anomalies in precipitation in Indonesia and the eastern Amazon, which are typically observed during La Niña events, may have driven increased emissions from tropical wetlands [*Dlugokencky et al.,* 2009; *Nisbet et al.,* 2014]. Another possible contribution to this increase in CH_4_ mole fraction is an increase of anthropogenic emissions mainly in Southeast Asia [*Houweling et al.,* 2014].

Wetlands are the largest contributor to global CH_4_ emissions and tropical South America and Africa dominate these emissions [*Kirschke et al.,* 2013]. Tropical South America shows the largest regional discrepancy between top‐down (17–48 Tg CH_4_ yr^−1^) and bottom‐up (39–92 Tg CH_4_ yr^−1^) wetland emissions [*Kirschke et al.,* 2013], indicating that emissions in this region remain uncertain. The Amazon Basin hosts the biggest humid tropical forests, and around 20% of its area is seasonally flooded [*Junk*, 1993]; thus, it is an important region for global CH_4_ emissions. Given the importance of CH_4_ as a greenhouse gas and its recent unanticipated and not entirely understood global atmospheric increase, it is of interest to analyze seasonal and interannual variability of tropical CH_4_ records and its controls. Here we analyze the longest existing CH_4_ record above tropical land. This is the record of regularly measured vertical profiles from 300 m above ground level to 4.5 km above sea level near Santarém (site code SAN) from 2000 to 2013, which is a follow‐up on the analysis published by *Miller et al.* [2007]. We expect the SAN CH_4_ record to be substantially influenced by wetland emissions and that it may tell us something about the sensitivity of these fluxes to changes in climate. Since variations in wetland emissions are thought to dominate the year‐to‐year variability in global surface emissions [*Kirschke et al.,* 2013], long term measurements can reveal possible relationships with precipitation and temperature variability.

In section 2 we will describe our methodology, including the flux calculations. Section 3 describes our results derived from the observations and discusses seasonality and interannual variability in the emissions from this period. Finally, we conclude the study in section 4.

## 2 Methodology

### 2.1 Air Sampling

**Figure 1:**
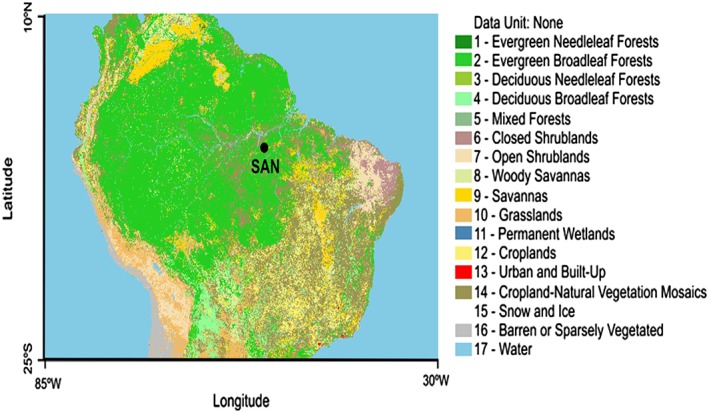
Land cover of South America from Large‐Scale Biosphere‐Atmosphere regional land cover map derived from the advanced very high resolution radiometer satellite, 1 km, version 1.2 (International Geosphere‐Biosphere Programme) (obtained from <http://webmap.ornl.gov/wcsdown/index.jsp>. The black dot shows the SAN location.

Vertical air profiles were sampled regularly from December 2000 onward over the Tapajos National Forest, Pará State, Brazil (2.86°S, 54.95°W), located approximately 70 km south of the city of Santarém (Figure 1). From 2000 to 2006, profiles were sampled on average once per month (see *Miller et al.* [2007] for details), and, starting in 2007 profiles were measured twice per month. Here we present the full record up to December 2013. SAN is located in the Amazon Basin approximately 700 km from the Atlantic coast, and its area of influence is covered by humid forest, savannas, degraded forest, and grasslands (caatinga) (Figure 1) [*Gatti et al.,* 2014]. The city of Belém, Pará State (population 2 million), also lies upwind from SAN.

Air samples were collected using a two‐component portable semiautomatic collection system, consisting of a first unit with two compressors and rechargeable batteries and a second unit with 17 borosilicate glass flasks of 700 mL each connected by tubing and valves which are opened and closed by a microprocessor. The microprocessor also records ambient temperature, pressure, humidity, coordinates, and time using GPS and temperature and relative humidity sensors connected to the compressor unit. Some of these variables (ambient temperature, pressure, and humidity) have been measured only from 2007 onward. These units were installed on board a small aircraft (Cessna 206) to sample ambient air connected to a tube connected to the outside of the right‐wing vent of the aircraft. The pilot initiates the sampling process once the top flight level has been reached. The samples were generally taken between 12:00 and 13:00, local time, when the boundary layer tends to be well mixed. During this time the profiles integrate fluxes from large regions [*Gatti et al.,* 2014].

### 2.2 Measurements

Between 2000 and 2003, we measured a smaller number of profiles compared with the other years, and these profiles were made mainly during the wet season, due to logistical problems. Also, these samples were analyzed in the laboratory of NOAA/ESRL (National Oceanic and Atmospheric Administration/Earth System Research Laboratory) in Boulder, USA. Since 2004, samples were analyzed at the Institute of Energy and Nuclear Research (IPEN), São Paulo, Brazil, using a measurement system for flask analysis that is a near replica of that used at NOAA. The CH_4_ analysis system uses FID (Flame Ionization Detector) chromatography (HP 6890 Plus) with a 198 cm, 3/16″ O.D. precolumn of length (Silica Gel 80/100 mesh) and a 106 cm × 3/16″ O.D. analytical column (Molecular Sieve 5A 80/100 mesh) and a 12 mL sample loop. This system also uses a 10‐port valve to inject the sample loop to precolumn, then just after the CH_4_ gas arrives to the column, the 10 port valve turns and starts a back flush in the precolumn to remove other gases, separating the CH_4_ from the rest of the air sample. The carrier gas used in this system is nitrogen with less than 0.1 ppm impurity. The system is highly calibrated using air from high pressure cylinders obtained from NOAA, whereby reference air is introduced before and after each sample. The accuracy and precision of our analysis system in Brazil is similar to that of the analysis system at NOAA [*Miller et al.,* 2007], with precision of 1.5 ppb. From 2004 onward, the number of profiles measured per year increased with regular measurements made during both wet and dry seasons. An exception is the year 2005, when we measured profiles again only during the wet season.

In this study we also use atmospheric greenhouse gas measurements of air from two stations of the NOAA Global Greenhouse Gas Reference Network: Ascension Island (ASC, 7.92°S, 14.42°W) located in the southern tropical Atlantic and from Ragged Point Barbados (RPB, 13.17°N, 59.43°W) located in the Caribbean. At those two stations, surface air is sampled using 2.2 L glass flasks and pumping units which fill the flasks to a pressure of about 120 kPa [*Conway et al.,* 1994]. Filled flasks are then sent to and analyzed for greenhouse gas dry air mole fraction levels at the NOAA/GMD laboratory in Boulder, Colorado, USA.

In this study we are combining measurements from two different laboratories, IPEN and NOAA, so accuracy is an important factor to be sure that observed CH_4_ mole fraction gradients between NOAA's and IPEN's sites do not include artifacts resulting from calibration differences between these two laboratories. The interlaboratory compatibility between IPEN and NOAA is better than 1 ppb as determined from colocated sampling at Natal on the east coast of Brazil (0.4 ± 3.2 ppb) and from a WMO sponsored “round‐robin” comparison of high pressure cylinders (0.7 ± 1.0 ppb). In order to further assess both the accuracy and long‐term repeatability of the CH_4_ measurements, previously calibrated tanks were measured as unknowns on the IPEN system on a regular basis. The measurements were made with two cylinders (“target tanks”) with natural air, calibrated previously by NOAA. These cylinders were analyzed 20 times with an interval of 60 or 15 days, depending on the cylinder over a period of more than 10 years. The results of these target tanks show long‐term repeatability (one sigma) of 1.5 ppb and a bias of 1.25 ppb.

As demonstrated above, measurements at both IPEN and NOAA are both tightly linked to the WMO X2004 CH_4_ mole fraction scale. WMO recommends compatibility for well‐mixed background air for CH_4_ a difference less than 2 ppb, and it is clear that this level is not being exceeded between NOAA and IPEN.

### 2.3 Region of Influence

**Figure 2:**
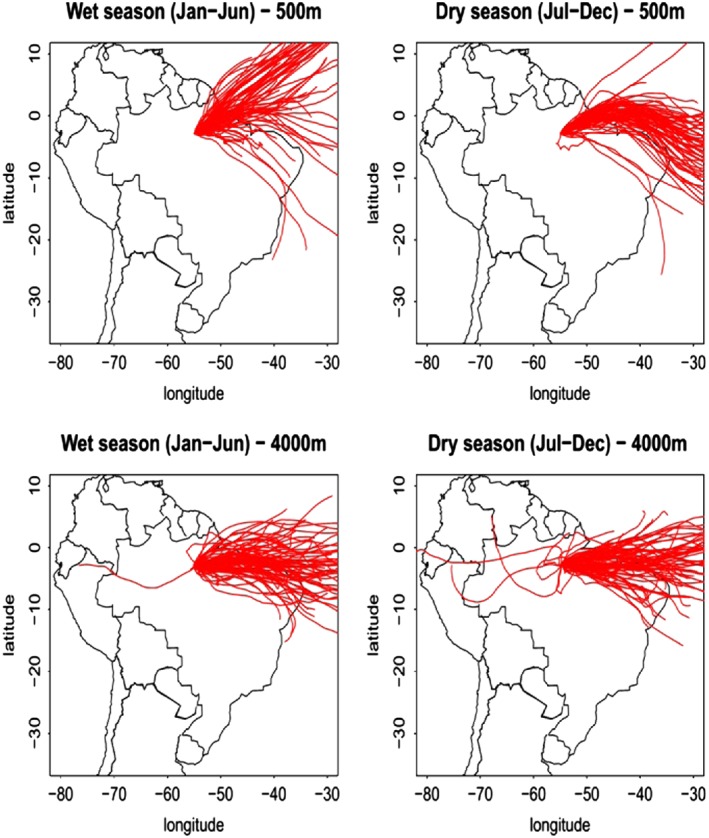
Back trajectories arriving at SAN at 500 and 4000 m above sea level for all vertical profiles sampled between December 2000 and December 2013, during the wet and dry season.

We are interested in the information about land‐atmosphere fluxes contained in the mole fractions of the vertical profiles measured at SAN. Although the ocean is part of the region of influence, oceanic emissions are not considered in this calculation, because we expect oceanic CH_4_ fluxes to be negligible compared to land fluxes. *Rhee et al.* [2009] estimate global oceanic emissions of CH_4_ to be 0.6–1.2 Tg yr^−1^. To isolate the land influence on the data we focus on differences between air entering the continent and SAN, ΔX = X_SAN_‐X_bg_. Here X is CH_4_ mole fraction, X_SAN_ is CH_4_ measured at SAN, and X_bg_ is CH_4_ of background air entering the basin at the coast. To understand which fluxes contribute to ΔX, we have calculated air mass back trajectories using the HYSPLIT trajectory model [*Draxler and Rolph,* 2013, http://ready.arl.noaa.gov/HYSPLIT_traj.php], with GDAS meteorological data (1° resolution), see Figure 2.

Air masses arriving at SAN enter the continent predominantly at the Brazilian northeastern coast (Figure 2). SAN back trajectories show some variability with varying altitude. At higher levels (4000 m) air mass trajectories cover a tighter angle in relation to the equator compared with the lower levels (500 m) and receive less influence from the Northern Hemisphere. In addition, trajectory directions vary somewhat seasonally, although mainly close to the surface, where wet season trajectories often have a more northerly component while dry season trajectories are more zonally directed and sample more Southern Hemisphere air. This seasonality in direction results from the seasonally varying position of the ITCZ (Intertropical Convergence Zone). During Northern Hemisphere summer its position is to the north of the equator, about 14°N during August and September, while during Southern Hemisphere summer its position is slightly south of the equator, around 2°S during March and April [*Cavalcanti et al.,* 2009].

**Figure A1:**
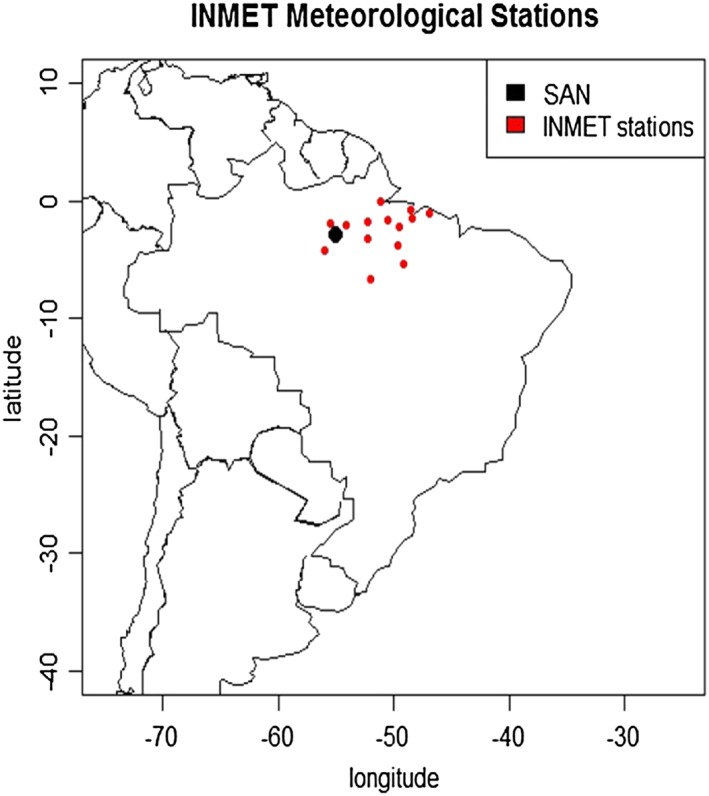
INMET meteorological stations used in this study and SAN location.

We use precipitation and temperature data from 14 stations located upwind of SAN (Figure A1). These data cover the period from 2000 to 2013 and are taken from the historical database of Instituto Nacional de Meteorologia [INMET‐ http://www.inmet.gov.br/portal/index.php?r=bdmep/bdmep].

### 2.4 CH_4_ Flux Estimation

We use a simple column budgeting technique to estimate CH_4_ fluxes following *Miller et al.* [2007] which is similar to the approach of *Chou et al.* [2002]. The difference between the methane column content at SAN and the coast is due to the sum of fluxes along the air parcel path. Thus, the net methane flux F_CH4_ in units of (g CH_4_ m^−2^ s^−1^) along the air mass path is as follows:

(1) $$F_{{CH}_{4}}=\int_{\text{surface}}^{4.4km}\frac{\left(CH_{4,SAN}\left(z\right)-CH_{4,bg}\right)}{t\left(z\right)}dz$$

Here *CH_4_* is methane concentration in units of (g CH_4_ m^−3^), *z* is height above ground (m), and *t(z)* is air mass traveltime (s) from the coast to the site and height z (m) above ground. The CH_4_ concentration is calculated from the measured dry air mole fraction X_CH4_ (mol CH_4_ mol air^−1^) as

(2) $${CH}_{4}\left(z\right)=\mu_{{CH}_{4}}\cdot n_{\text{air}}\left(z\right)\cdot X_{{CH}_{4}}=\mu_{{CH}_{4}}\cdot \frac{p_{\text{air}}\left(z\right)}{R\cdot T\left(z\right)}\cdot X_{{CH}_{4}}$$

where $\mu_{{CH}_{4}}$ is molar mass of methane (16 g (molCH_4_)^−1^), *n*_air_(*z*) is air number density ((mol air) m^−3^) at height z above ground, *p*_air_(*z*) is air pressure (atm), *T*(*z*)(K) is temperature, and *R* = 8.205 × 10^−5^ (m^3^ atm K^−1^ mol^−1^) is the ideal gas constant. Pressure p_air_ is assumed to change with height according to *p*_air_(*z*) = *p*_air_(0)*e* ^− *z*/H^ (atm) where *H* = 7000 m is the scale height of the atmosphere and p_air_(0) = 1 (atm). Temperature is assumed to decrease with height either as measured or, if measurements of temperature are missing, following

(3) $$T\left(z\right)=T_{srf}+\Gamma \cdot \left(z-z_{srf}\right)$$

where T(z) is temperature at height z above ground, T_srf_ is the mean surface temperature, z is height above sea level, z_srf_ is height of the surface, and Γ = −6.5 K km^−1^ is the average temperature lapse rate value at SAN [*Miller et al.,* 2007].

To estimate the traveltime *t* of air masses from the coast to SAN we calculated back trajectories for altitudes from 500 m to 4500 m in steps of 500 m using HYSPLIT, and altitudes of air samples were associated with the closest level in the vertical.

**Figure A2:**
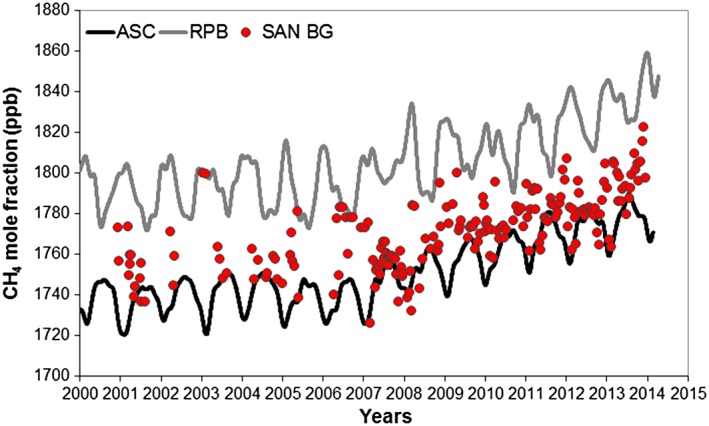
CH_4_ time series from ASC, RBP, and SAN BG (background mole fractions).

To calculate the flux using this method an estimate of the CH_4_ mole fraction of air entering the continent from the sea, CH_4_,_bg,_ is needed. As shown in Figure 2 air enters the Amazon Basin mainly from the Atlantic Ocean. We expect that depending on the season and position of the ITCZ, incoming air will have larger or smaller contributions of Southern versus Northern Hemisphere air. We therefore estimate background mole fractions (Figure A2) as a mixture of Northern Hemisphere and Southern Hemisphere air and estimate the mixing fractions f using SF_6_ as a tracer of Northern versus Southern Hemisphere air [*Miller et al.,* 2007]. As end‐members for the linear mixing model we use the NOAA background site records of Ragged Point Barbados (13.17°N, 59.43°W; Northern Hemisphere air) and Ascension (ASC, 7.92°S, 14.42°W; Southern Hemisphere air). Thus, we estimate CH_4_, _bg_ as follows:

(4) $$CH_{4,bg}=f^{ASC}C{H_{4}}^{ASC}+\left(1-F^{ASC}\right)C{H_{4}}^{RPB}$$

where

(5) $$f^{ASC}=\left(S{F_{6}}^{SAN}-S{F_{6}}^{RPB}\right)/\left(S{F_{6}}^{ASC}-S{F_{6}}^{RPB}\right)$$

SF_6_ is suited for this purpose because it exhibits a distinct Northern to Southern Hemisphere difference. This is because the sources of SF_6_ are mainly in the Northern Hemisphere, and there are no significant SF_6_ sources in the Amazon [*Emissions Database for Global Atmospheric Research (EDGAR*), 2009]. SF_6_ is emitted mainly by leakage from electrical power distribution stations where it is used as an insulator and is thus closely linked to energy consumption [*Maiss et al.,* 1996; *Gloor et al.,* 2007]. Eighty‐five percent of the SF_6_ mole fraction values measured at SAN are indeed in between the values measured at ASC and RPB. SF_6_ data from the Natal intercomparison between IPEN and NOAA show an average difference of 0.00 ppt between 2010 and 2013, with a maximum average difference of 0.02 ppt; cylinder round‐robin intercomparisons show an average difference from NOAA of 0.01 ppt. In order to test the sensitivity of our results to biases between IPEN and NOAA SF_6_ observed in this period, we shifted IPEN results in 2008 and 2009 by 0.01 ppt. Results showed that this shift in SF_6_ affected CH_4_ fluxes only by 4–5 %, demonstrating that potential bias between the two networks is not a significant source of error and that SF_6_ is a suitable air mass tracer. Monte Carlo error propagation analysis (more details in section 2.6) showed variability in 13 years of CH_4_ mean flux of 13%. So the possible influence in CH_4_ fluxes by the SF_6_ bias is significantly lower than the CH_4_ flux uncertainty caused by all possible sources of error.

### 2.5 CH_4_ Emissions From Biomass Burning

**Figure A3:**
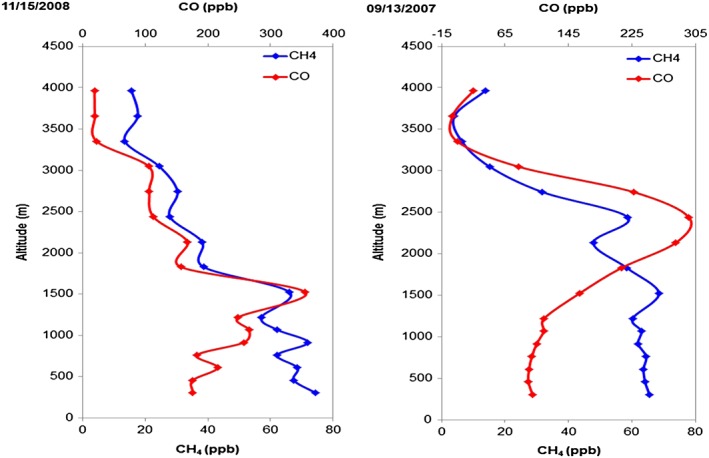
CH_4_ and CO vertical profiles to different days: 15 November 2008 and 13 September 2007 illustrating the profiles used in the calculation of the emission ratio CO/CH_4_.

Biomass burning emits CH_4_ and also CO. Although the CH_4_ to CO ratio varies depending on the nature of fire, this ratio permits an approximate estimate of CH_4_ emissions caused by biomass burning as F_CH4,BB_ = (1/r_CO:CH4_) × F_CO_ provided F_CO_ is known. Here F_CO_ is the CO flux in g CO m^−2^ s^−1^ estimated analogously to the CH_4_ flux from each profile and r_CO:CH4_ = 6.7 ± 1.9 ppb CO/ppb CH_4_ = (28/16)*(6.7 ± 1.9) g CO/g CH_4_ is the mean (and one sigma variability) emission ratio estimated based on the profile data. To estimate the r_CO:CH4_ ratio we selected only profiles during the dry season, in which, after subtraction of a CO background, a plume (a large positive anomaly in ΔCO mole fraction) from biomass burning was clearly identifiable in the profile (Figure A3). We furthermore only used such events for which the plume was above 1.5 km height, to avoid influence from local sources. We found 12 profiles which fulfilled these criteria over 13 years.

CO produced during biomass burning decreases with time due to oxidation by OH. In the tropical dry season the OH mole fraction can be as high as 2.8 × 10^6^ molecule cm^−3^ [*Spivakovsky et al.,* 2000] implying a CO lifetime of about 20 days [*Demore et al.,* 1997]. Due to this oxidation, we correct the emission factor. Considering that the SAN region has a mean transit time of 2.8 days from the Brazilian coast, we estimate a reduction of the true emission ratio of 14%, resulting in r_CO:CH4_ = 7.4 ± 1.8 ppb CO/ppb CH_4_.

To correctly remove the biomass burning flux from the total CH_4_ flux it is necessary to include the effect of a natural CO flux from soil [*Conrad and Seiler*, 1985] and as a byproduct of isoprene emissions by trees [*Kuhn et al.,* 2007]. In order to estimate this biogenic CO flux, we used the observation that the total CO flux of 26.7 mg CO m^−2^ d^−1^ calculated for SAN is approximately constant between March and June [*Gatti et al.,* 2010], although it is likely that in the dry season biogenic CO emissions are somewhat greater than during the rainy season, due to increased emissions of isoprene [*Trostdorf et al.,* 2004] and the increase of OH [*Spivakovsky et al.,* 2000]. Here, for simplicity, it is assumed that the period of stable biogenic CO persists throughout the year [*Gatti et al.,* 2010].

Our emission ratio is similar to previously published values. *Yokelson et al.* [2007] estimated a ratio of 10.2 ± 0.1 ppb CO/ppb CH_4_ using measurements taken with an airplane during the 2004 dry season in the Amazon. *Andreae and Merlet* [2001] published an emission ratio for tropical forests of 8.7 ± 1.3 ppb CO/ppb CH_4_, and *Akagi et al.* [2011] estimated a ratio of 10.5 ± 1.7 ppb CO/ppb CH_4_ for tropical forests. Approximately 46% of the area upwind of SAN is covered by forest. Thus, fires from nonforest areas that influence vertical profiles measurements at SAN could explain these differences in CO:CH_4_ ratios. If we use a ratio of 10 ppb CO/ppb CH_4_, as previously published, the fire flux determined from SAN data would be reduced by 26%. Additionally, to estimate the contribution of anthropogenic emissions of CH_4_ we used values from EDGAR database version 4.2.

### 2.6 Uncertainty Analysis

The uncertainty of our approach was estimated by error propagation with Monte Carlo randomization. We took into account the uncertainty in the background concentration and the uncertainty in air parcel traveltime, and for separation of total fluxes in fire and land vegetation fluxes unrelated to fire, we account for the uncertainty in r_CO:CH4_. In the calculation of the background values, we account for the more significant (~0.5%) measurement uncertainty for SF_6_. We assume uncertainties of back trajectory traveltimes to be normally distributed with a standard deviation of 0.3 day (about 10%) for SAN. Uncertainties of background mole fractions CH_4_,bg (equation (4)) vary seasonally and are derived by propagating the 0.5% uncertainty in median SF_6_ values in equation (5), where uncertainties from SF_6_^ASC^ and SF_6_^RPB^ come from the standard deviation of the residuals to curve fits [*Thoning et al.,* 1989] (using a short‐term residuals smoother of about 150 days) to CH_4_ and SF_6_ observations. Uncertainty in r_CO:CH4_ was normally distributed with a standard deviation of 1.8 ppb CO/ppb CH_4._ We calculated the annual mean total, biogenic, and biomass burning fluxes, and their uncertainties for each set of randomly perturbed profiles for the 13 year period.

We also used bootstrapping of monthly mean flux to estimate annual mean uncertainties, for which 95% confidence intervals are slightly smaller than the uncertainty estimates calculated using Monte Carlo randomization. We therefore report here the larger fluxes uncertainties from the Monte Carlo approach.

## 3 Results and Discussions

### 3.1 Vertical Structure of CH_4_ Profiles and Land Surface Flux Signal

**Figure 3:**
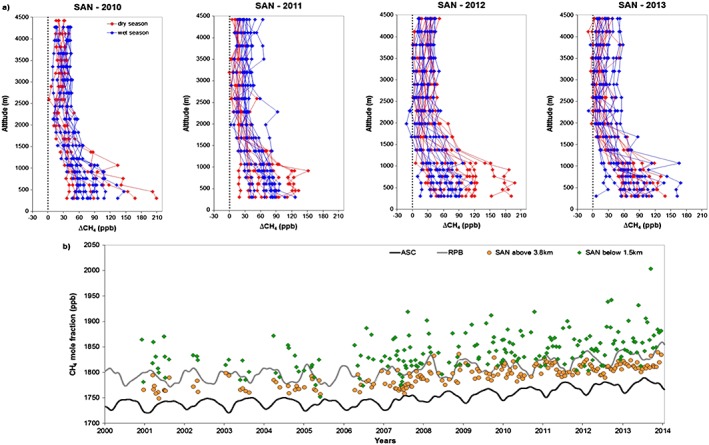
(a) ∆CH_4_ (in situ minus background mole fraction) vertical profiles at SAN measured during wet and dry seasons for the years 2010–2013. (b) CH_4_ time series of ASC and RPB and the SAN mean mole fraction above 3.8 km and below 1.5 km. Measurements uncertainty is 1.5 ppb.

The difference between the mole fractions at the sampling site and background mole fractions is a simple way to observe terrestrial sources and sinks and is directly related to terrestrial CH_4_ fluxes [*Miller et al.,* 2007]. The lower levels of the profiles (within the planetary boundary layer, below around 1.5 km) are the parts most influenced by the process that occur at the surface. SAN vertical profiles indeed show enhancements in these lower altitudes in comparison with the higher altitudes, indicating significant emissions in the eastern Amazon Basin, during the whole year (in both wet and dry season). At higher altitudes, CH_4_ levels are well mixed and are thus likely representative of the CH_4_ background air entering the basin (Figure 3a). Note, however, that in our quantitative analysis background is represented as a linear combination of ASC and RPB ASC and RPB.

Indeed, mean mole fractions above 3.8 km (altitudes with less variability which represent the free troposphere) are almost always between those at ASC and RPB, like the estimated BG mole fractions. The differences in mole fraction between the lower and upper parts of the profile (below 1.5 km and above 3.8 km) are thus caused by surface sources and sinks (Figure 3b). The higher mean mole fractions below 1.5 km compared with the mean above 3.8 km is a clear indication that this region of the Amazon Basin is a substantial source of CH_4_ during the whole year. For the 194 profiles analyzed here, the annual mean difference between the free troposphere (above 3.8 km) and below 1.5 km at SAN is 49.0 ± 33.7 ppb. The mean vertical gradient in the wet season (January to June) is 42.5 ± 27.0 ppb, and the mean gradient in the dry season (July to December) is 56.2 ± 38.8 ppb, suggesting that emissions are higher in the dry season, assuming similar air parcel travel times, t(z) over land in each season.

### 3.2 Annual Mean and Seasonal CH_4_ Fluxes

**Figure 4:**
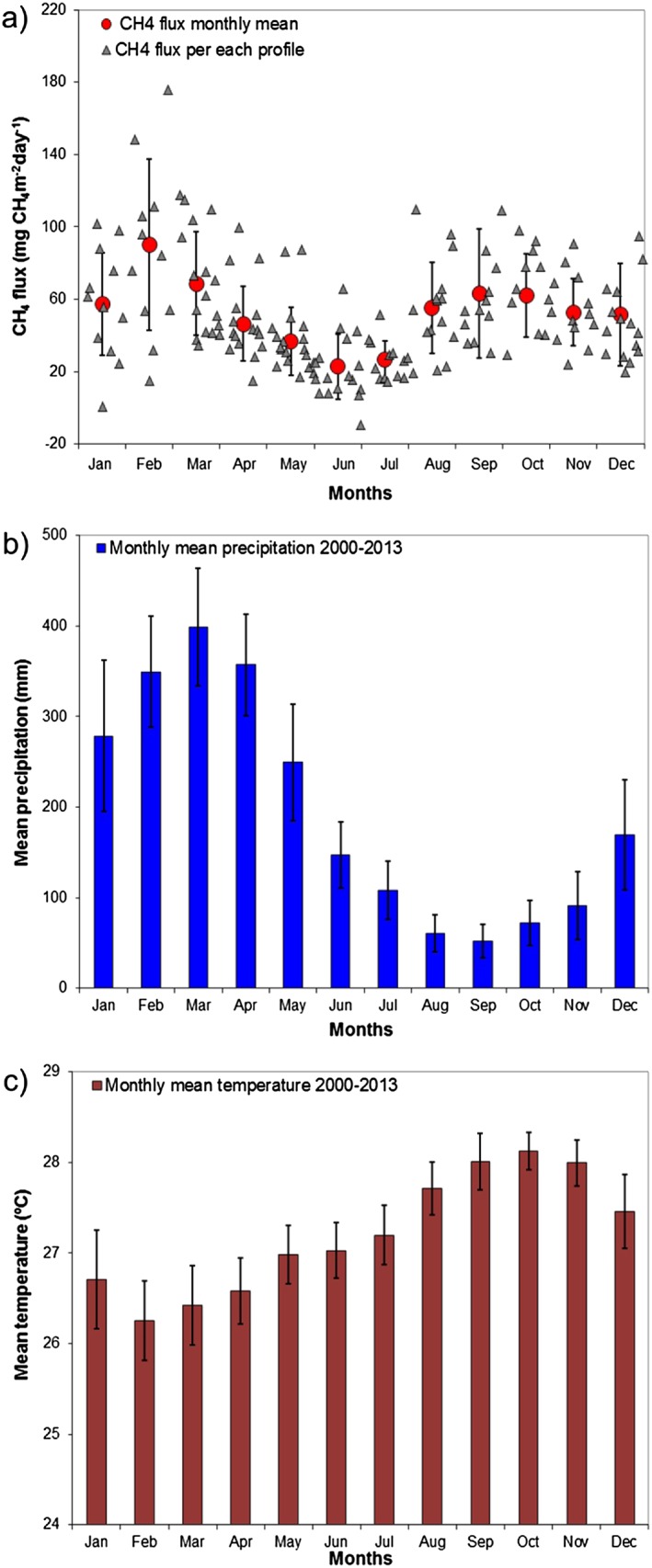
(a) CH_4_ flux per profile, between 2000 and 2013, and the monthly mean for all years. (b) Mean precipitation of 14 INMET meteorological stations located upwind of SAN. (c) Mean temperature at the same 14 INMET stations. Error bars represent the standard deviation of the monthly means.

Although vertical gradients suggest qualitatively a substantial CH_4_ source, we now estimate the CH_4_ flux quantitatively for eastern Amazonia by using the column budgeting technique described in section 2.4. Figure 4 shows climatological monthly fluxes for all 13 years for the area upwind of SAN. The fluxes exhibit a clear seasonality, with two periods of elevated emissions: first in the beginning of the year, between January and March with a mean flux of 71.9 ± 34.7 mg CH_4_ m^−2^ d^−1^ (where 34.7 is the one‐sigma standard deviation of monthly fluxes) and second between August and December with a mean flux of 57.0 ± 26.2 mg CH_4_ m^−2^ d^−1^. A minimum in emissions was observed in June with a mean flux between April and July of 33.1 ± 17.0 mg CH_4_ m^−2^ d^−1^, and the mean annual flux for this region is 52.8 ± 6.8 mg CH_4_ m^−2^ d^−1^ (where 6.8 is the 95th percentile confidence interval of annual fluxes calculated using the Monte Carlo error propagation).

*Miller et al.* [2007] found a mean flux of 35 ± 23 mg CH_4_ m^−2^ d^−1^, between 2000 and 2006 at SAN. Although our results cover a longer period, this difference can be explained in part by the difference in t(z) used to estimate fluxes. *Miller et al.* [2007] used a mean traveltime of 2 days throughout the profile based on wind speed climatologies, while we use back trajectory times calculated with the HYSPLIT model for each individual profile and for each measurement height of the profile. Using a mean time of 2 days like *Miller et al.* [2007], we found a reduction of 15% of our total annual mean flux. Until 2006 SAN profiles were measured mainly during the wet season, and we find higher emissions at SAN during dry season.

In addition to this general seasonality, fluxes showed variability for different profiles from the same month. The variability is generally higher during January to March than in the other months, with the maximum variability and maximum mean emissions in February (Figure 4a). Flux variability tends to decrease throughout the subsequent months, with a second increase in August until December. This seasonality observed in CH_4_ fluxes, with higher variability in beginning of the year and lower variability in the subsequent months, is very similar to that observed earlier for CO_2_ by *Gatti et al.* [2010].

**Figure 5:**
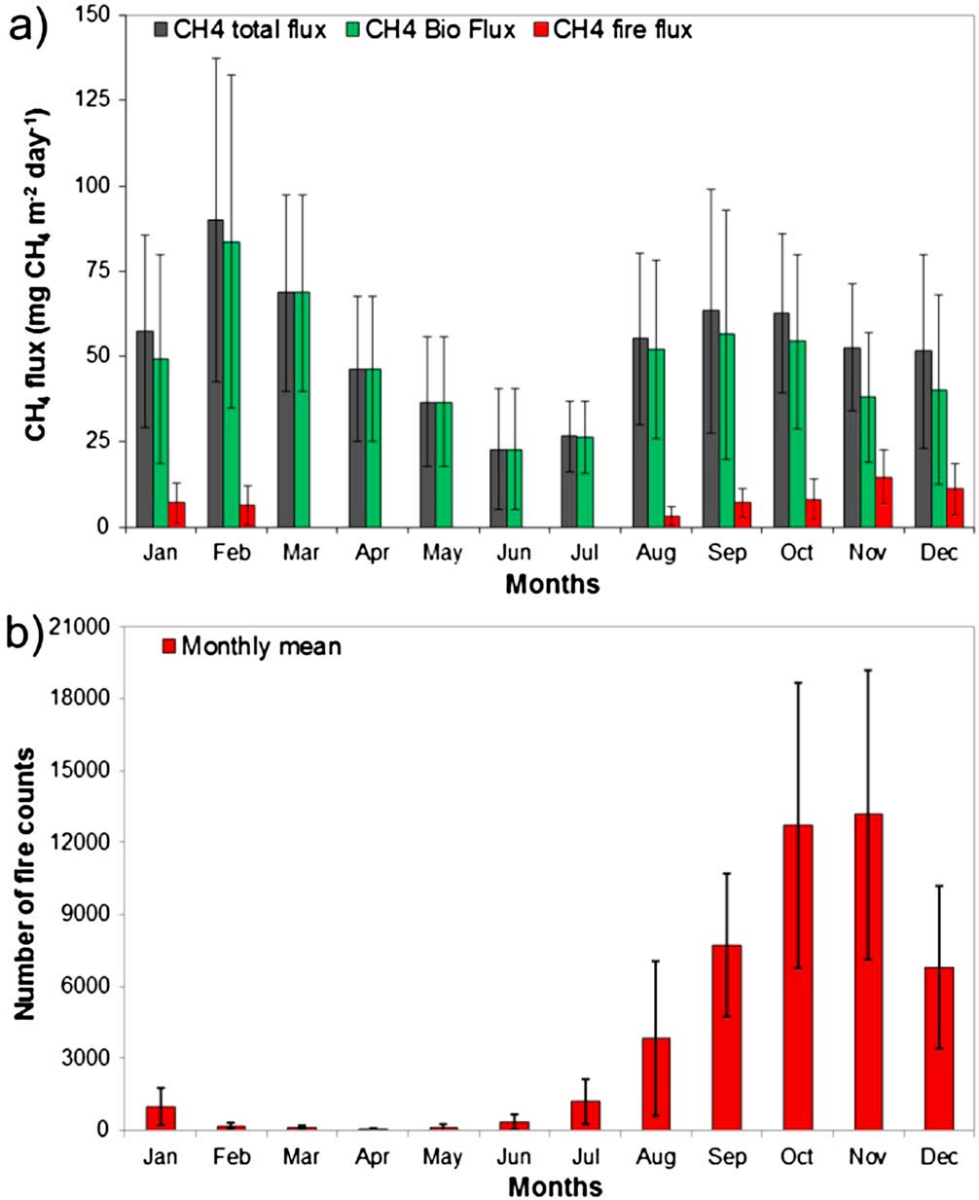
(a) CH_4_ total mean flux, CH_4_ biogenic mean flux and CH_4_ mean flux from biomass burning. Error bars represent the standard deviation of the monthly means. (b) Monthly mean of fire counts during 2000 to 2013, obtained from CPTEC/INPE (http://www.dpi.inpe.br/proarco/bdqueimadas/), for the area between SAN and Brazilian coast. Error bars represent the standard deviation of the monthly means.

**Figure A4:**
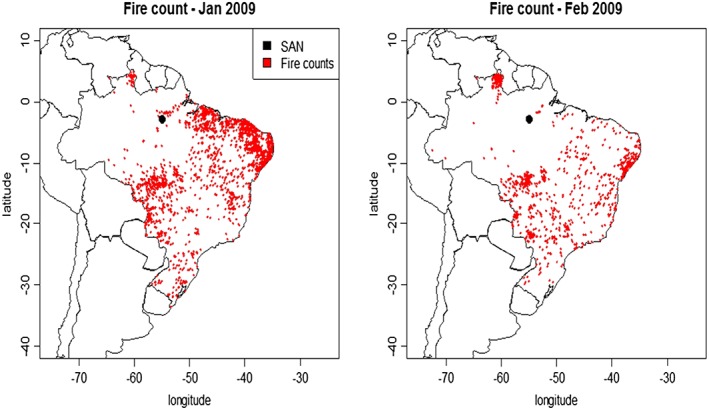
SAN location and the fire counts during January and February 2009, obtained from CPTEC/INPE (http://www.dpi.inpe.br/proarco/bdqueimadas/).

The total CH_4_ flux is the result of wetland, biomass burning, and anthropogenic emissions. Using the biomass burning CO:CH_4_ emission ratio, we subtracted the biomass burning fluxes, F^BB^_CH4_ (see section 2.5) from the total flux. The result of the subtraction of the biomass burning emission from the total emission is denoted “biogenic” flux and includes natural emissions from wetlands and anthropogenic emissions. Figure 5 shows climatological monthly means of total, biogenic, and biomass burning CH_4_ fluxes. We find that the region upwind of SAN had an annual mean biogenic flux, during the years 2000 and 2013, of 47.7 ± 4.8 mg CH_4_ m^−2^ d^−1^ (where 4.8 is the 95th percentile confidence limit of annual fluxes calculated using the Monte Carlo error propagation), and an annual mean flux from biomass burning of 4.9 ± 0.7 mg CH_4_ m^−2^ d^−1^, indicating that only approximately 9% of the total annual CH_4_ flux estimated for this region is from biomass burning. During the months of high precipitation (January and February) we somewhat surprisingly occasionally observed profiles with high mole fractions of CO, indicating emissions from biomass burning, which represent 10% of total flux of this period. In the eastern part of the Amazon and in the Brazilian northeast coast, biomass burning was observed in these months (Figure A4). Figure 5b shows the monthly mean of fire counts for the region between the Brazilian coast and SAN. During the primary biomass burning season (August to December), we estimate a biogenic flux of 48.2 ± 27.0 mg CH_4_ m^−2^ d^−1^ and a biomass burning flux of 8.8 ± 5.6 mg CH_4_ m^−2^ d^−1^ (where 27 and 5.6 mg CH_4_ m^−2^ d^−1^ are the standard deviation of monthly mean fluxes), representing only 15% of the total CH_4_ flux in this period. We furthermore find that emissions from biomass burning in the region upwind of SAN increase gradually from July to November (Figure 5), as does the occurrence of fire counts. After removal of the biomass burning flux from the total flux substantial CH_4_ emissions remain, indicating that there are other significant sources with biogenic origin during the dry season.

Continuous higher emissions in the dry season without a clear biomass burning contribution are consistent with the observations of *Beck et al.* [2012], who found only a minor influence from biomass burning on the observed CH_4_ enhancements during the dry season and an excess CH_4_ of biogenic origin determined by isotope analysis.

Extrapolating the annual mean estimate of the biomass burning flux of 4.9 ± 0.7 mg CH_4_ m^−2^ d^−1^ to the whole year and the total forest area upwind of SAN (around 0.6 × 10^6^ km^2^), we obtain an emissions estimate of 1.0 Tg CH_4_/yr^−1^. An independent estimate of biomass burning emissions based on satellite imagery is available from the Global Fire Emissions Database version 4 (GFED4) (an updated version of the original GFED version 1 of *van der Werf et al.* [2010] with burned area from *Giglio et al.* [2013] boosted by small fire burned area [*Randerson et al.,* 2012]). Our biomass burning estimate is much larger than the GFED4 emission estimate which averages 0.02 Tg CH_4_/yr^−1^ over 2000–2013 (including all fire types). According to *Giglio et al.* [2013] burned area in persistently cloudy regions will be systematically underestimated. We thus have analyzed outgoing longwave radiation (OLR) [*Liebmann and Smith*, 1996] for this region as an indicator of cloud cover. We have also examined emission factors for CH_4_ used by GFED (emissions factors from *Akagi et al.* [2011]) compared to the CO:CH_4_ ratio based on our data (7.4 ppb CO/ppb CH_4_) and found that this cannot explain the emissions discrepancy. The OLR data reveal persistent and dense cloud cover over the SAN upwind area during the Amazon fire season. We therefore attribute the large difference in the emissions estimates to a lack of visibility of fires from space in this region and during this period, which causes the GFED estimate to be much too low.

The region upwind of SAN had a mean annual flux of 52.8 ± 6.8 mg CH_4_ m^−2^ d^−1^. *Melack et al.* [2004] estimated an emission of CH_4_ from flooded areas of the Amazon Basin of 22 TgC yr^−1^ (equivalent to 29 Tg CH_4_ yr^−1^), but their analysis did not include much of the eastern basin upwind of SAN. If we divide the *Melack et al.* [2004] emission by the total area of the Amazon Basin (5 × 10^6^ km^2^) we obtain a flux of 16 mg CH_4_ m^−2^ d^−1^. Comparing this estimated flux from wetlands with the flux obtained in the SAN region suggests that the eastern part of the Basin may have significantly larger fluxes than the rest of Amazon. In terms of anthropogenic fluxes, the EDGAR database estimates total anthropogenic emissions of 5.7 mg CH_4_ m^−2^ d^−1^ for the area upwind of SAN. These emissions include those from enteric fermentation (52%), agricultural emissions (4%), leaks from gas and oil production and distribution (7%), waste (21%), energy (8%), industrial process (2%), fugitive from solid (4%), and residential and transport (1%). Total anthropogenic emissions represent only 11% of the SAN‐based total flux, although some of the emissions (energy, industrial, and transport are captured to some extent by our CO correlation method). Thus, by deduction, it is very likely that the main source of the high emissions in our study area is from seasonal and permanent wetlands.

### 3.3 Precipitation and Temperature Influence on CH_4_ Fluxes

As already mentioned, our CH_4_ data and fluxes exhibit a seasonality with the highest emissions in the beginning of the year (January until March) followed by a decline and another period with elevated emissions from August until December (Figure 4a). The first period of higher emissions occurs in the months during the wet season (Figure 4b). This suggests an important contribution from natural sources to the CH_4_ flux, specifically from seasonally flooded areas. The second period of higher emissions occurs during the dry season (August to December), during the period of higher temperatures (Figure 4c), where only 15% of this flux is from biomass burning. This result indicates the influence of wetland emissions during the dry season in the upwind region of SAN.

*Sawakuchi et al.* [2014] found that the Xingu and Tapajós rivers (both near SAN) have highest and second highest CH_4_ emissions, respectively, in comparison with other large rivers in the Amazon region and that all rivers had higher fluxes during low water levels with approximately 4 times more flux than during the higher water levels. Although fluxes from these rivers were high with an average flux of 95 ± 146 mg CH_4_ m^−2^ d^−1^ (from the Xingu river) and 39 ± 66 mg CH_4_ m^−2^ d^−1^ from the Tapajós River, the area covered by the rivers is extremely small (~1%) in comparison to the area influencing our profiles. Thus, when scaling this process by river area, we cannot explain our results by this mechanism. However, if we scaled the *Sawakuchi et al.* [2014] flux estimate by flooded area ‐ not just river area ‐ a large fraction of our air concentration based estimate could be explained.

**Figure 6:**
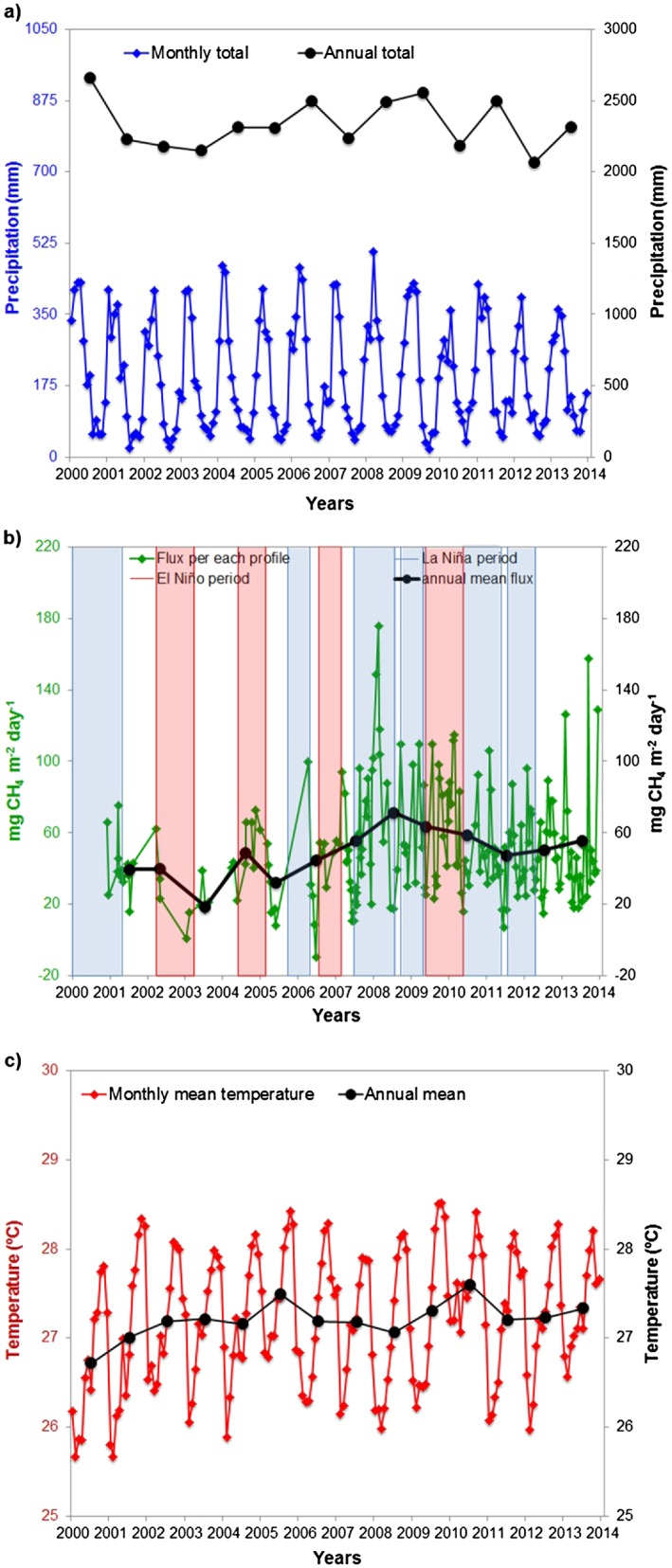
(a) Monthly mean and total annual precipitation from 14 INMET stations located upwind of SAN, (b) CH_4_ fluxes for each profile and annual mean fluxes, the bars represent La Niña and El Niño periods. Uncertainties of annual mean fluxes have been estimated using bootstrapping of monthly mean data, (c) monthly mean and annual mean temperature from 14 INMET stations located upwind of SAN.

**Figure 7:**
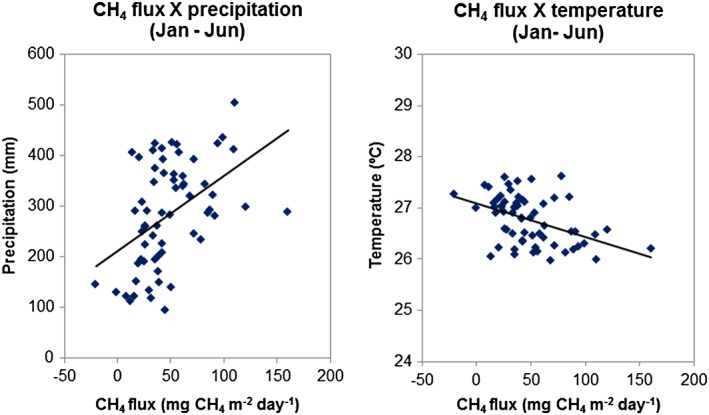
Correlation between CH_4_ flux monthly mean and monthly mean precipitation and temperature, between January and June. Precipitation and temperature are calculated as the mean of precipitation and temperature records measured at the 14 INMET meteorological stations located upwind to SAN.

**Figure A5:**
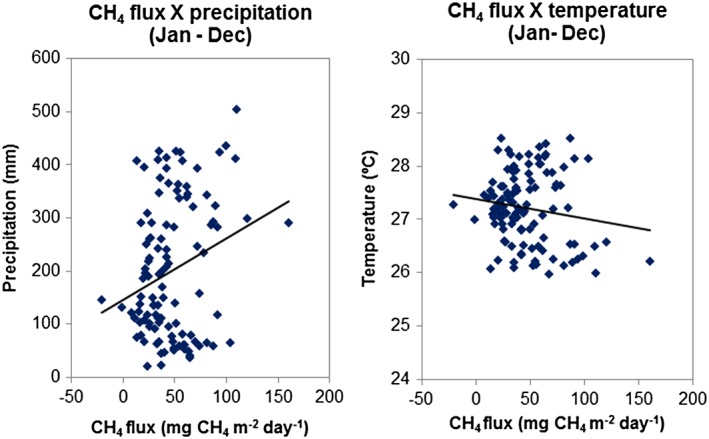
Correlation between the CH_4_ flux monthly mean and monthly mean precipitation and temperature from 14 INMET stations located upwind to SAN.

The CH_4_ fluxes and precipitation records show some relation between the two, while there does not seem to be a relation with temperature (Figure 6). In order to quantify this, we calculated a simple linear regression between CH_4_ flux and precipitation or temperature. Analyzing mean monthly CH_4_ biogenic fluxes and the monthly total precipitation and the monthly mean temperature, we found a weak correlation between monthly mean biogenic flux and precipitation (*r* ^2^ = 0.06, *p* value of 0.0048 (Figure A5)) while with temperature we found a weak and nonstatistically significant anticorrelation (*r* ^2^ = 0.03, *p* value of 0.0900 (Figure A5)). Excluding the months of July through December, to consider only the wet season period (Figure 7), we found a correlation between biogenic flux and precipitation of *r* ^2^ = 0.21 with a *p* value of 0.0001, and an anticorrelation with temperature of *r* ^2^ = 0.20 with a *p* value of 0.0030. Higher monthly mean temperatures occur during the dry season (July to December, Figure 4c). During this period the biogenic fluxes and temperature showed a correlation of *r* ^2^ = 0.19 with a *p* value of 0.0013. A multiple linear regression with precipitation and temperature with the flux showed a correlation of *r* ^2^ = 0.07 with a *p* value of 0.017 between January and December and a correlation of *r* ^2^ = 0.21 with a *p* value of 0.004 between January and June (wet season). Results showed weak but statistically significant correlations between CH_4_ fluxes and precipitation and temperature. These results suggest that other environmental factors besides precipitation and temperature are important controls on variability in methane emissions.

### 3.4 CH_4_ Flux Interannual Variation

The 13 years of measurements at SAN allow us to observe and to try to understand interannual variations in emissions. As already mentioned, previous studies [*Dlugokencky et al.,* 2009; *Nisbet et al.,* 2014] show that globally, the CH_4_ mole fraction increased from 2007 onward, after a relatively stable period between 1999 and 2006. One possible reason for this increase is an increase in tropical wetland emissions during La Niña periods in 2007 and 2008 [*Dlugokencky et al.,* 2009], for example, from Amazonia.

The time series of CH_4_ flux at SAN show interannual variation of ± 13 mg CH_4_ m^−2^ d^−1^ (one sigma) (Figure 6). Most specifically for the year 2008 we found the largest emissions with emissions 3 Tg CH_4_ higher than 2007 (when extrapolating CH_4_ flux to the forest area upwind of SAN), which occur mainly during the wet season. CH_4_ flux time series (Figure 6b) show that in the beginning of this year (between January and March and mainly in February) there were higher emissions in comparison with other years. Vertical gradients of these profiles showed a significant increase in mole fractions below 1.5 km, indicating significant regional emissions influencing these profiles.

**Figure 8:**
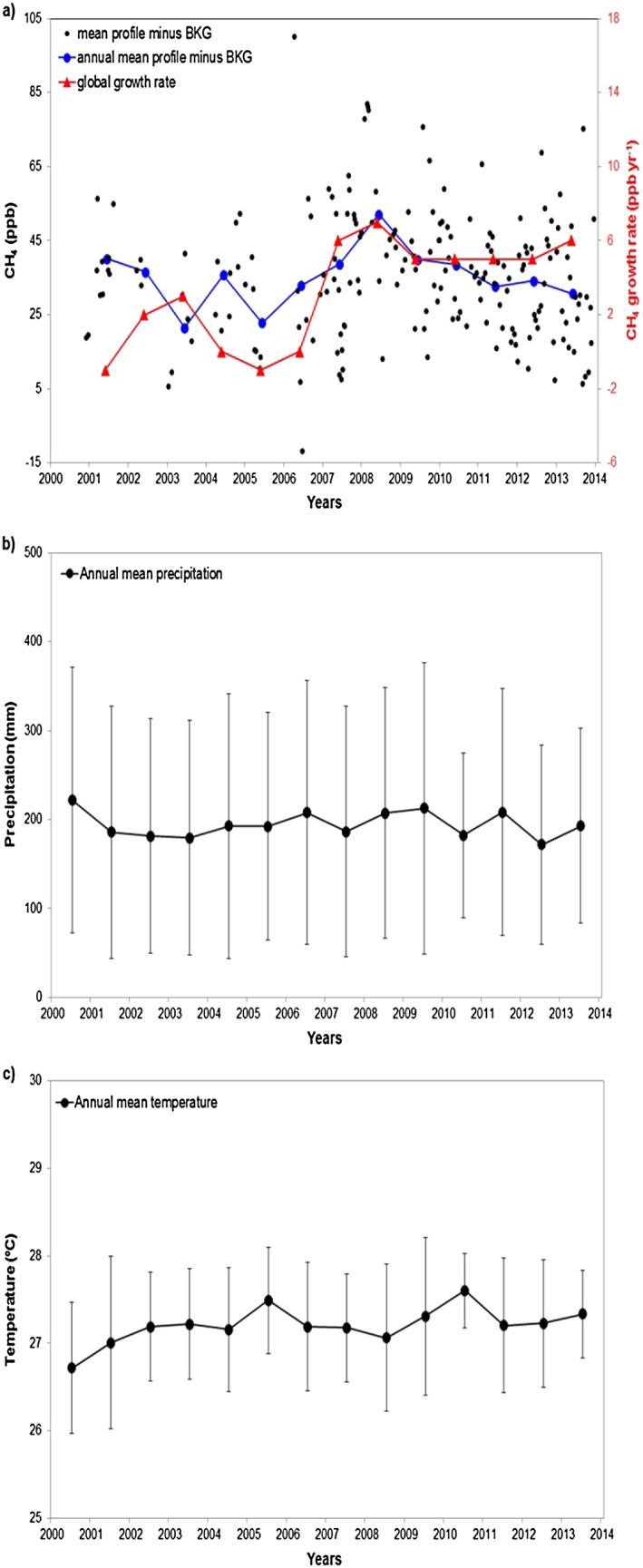
(a) Difference of the mean profile minus the BG mole fractions for each profile, the annual mean of this difference and the global CH_4_ growth rate, (b) Annual mean precipitation from 14 stations upwind SAN, (c) Annual mean temperature from 14 stations upwind SAN. Error bars represent the variability of the annual means for precipitation and temperature.

**Figure A6:**
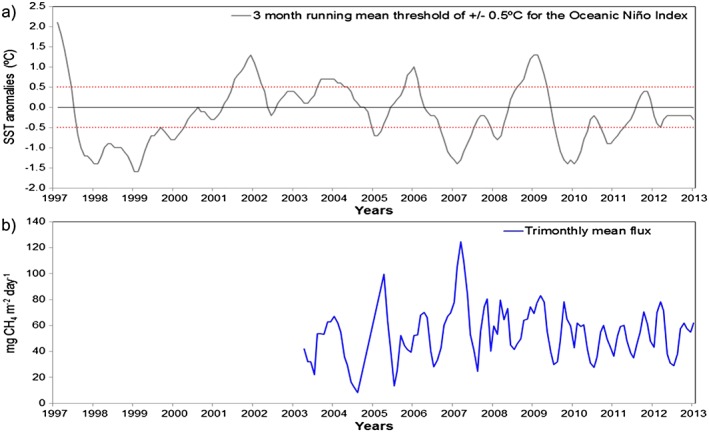
(a) Trimonthly threshold of ± 0.5°C for the Oceanic Niño Index (ONI) (3 month running mean of ERSST.v4 Sea Surface Temperature (SST) anomalies in the Niño 3.4 region, 5°N–5°S, 120°–170°W) that indicate the occurrence of the El Niño and La Niña (data available at <http://www.cpc.noaa.gov/products/analysis_monitoring/ensostuff/ensoyears.shtml>). (b) Trimonthly mean of total CH_4_ fluxes for SAN region.

Comparing the difference between mean profile mole fractions minus the background mole fractions in the SAN region with the global growth rate [*WMO, GAW, WDCGG,* 2014] (Figure 8), we observed a higher vertical difference during 2008 which is consistent with the global increase, but which is not observed during the other years. This result indicates that Amazonia may have contributed to the increase in CH_4_ global mole fraction during 2008, but not significantly afterward. Analyzing the threshold of ± 0.5°C for the Oceanic Niño Index (ONI) (3 month running mean of ERSST.v4 sea surface temperature anomalies in the Niño 3.4 region, 5°N–5°S, 120°–170°W that show the occurrence of the El Niño and La Niña events, Figure A6) indicates that the 1998–1999 and 2007–2008 La Niña were similar, but in 1998–1999 La Niña lasted longer than in 2007–2008. Another La Niña occurred in 2010–2012 and was similar to the 2007–2008 event. However, the regional SAN precipitation in these periods did not increase during La Niña periods, and we also did not find differences in the regional temperature. While analyzing the CH_4_ fluxes, we found higher emissions at SAN during the 2007–2008 La Niña (Figure A6), but this increase in CH_4_ emissions during La Niña was not observed during the second La Niña event (2010–2012). Thus, it is not possible to confirm a relationship between higher CH_4_ emissions and La Niña periods in eastern Amazonia. We also found no clear relation between this emission increase and changes in temperature or precipitation in 2008.

CH_4_ emissions after 2006 were around 44% higher (the mean annual flux between 2007 and 2013, 56.5 mg CH_4_ m^−2^ d^−1^) in comparison to the period 2000–2006 (with a mean annual flux of 39.4 mg CH_4_ m^−2^ d^−1^). It is important to highlight that until 2005 a small number of profiles were sampled in comparison with the other years, and these profiles were sampled mainly during the wet season. We did not find a difference in temperature or precipitation (or fire emissions) between these two periods that can be correlated with the difference in emissions.

## 4 Conclusions

The region between the Atlantic coast and SAN (around 1.3 × 10^6^ km^2^) was a large source of CH_4_ during the entire study period (2000–2013), with a CH_4_ annual mean flux of 52.8 ± 6.8 mg CH_4_ m^−2^ d^−1^. We find a clear seasonality in CH_4_ flux in this region of the Amazon Basin, with two periods of higher emissions: from January to March and from August to December. For the wet season, we find a weak but statistically significant correlation between precipitation and biogenic fluxes. For the dry season, a similarly weak yet statistically significant correlation was found with temperature. Natural sources, like wetlands, are likely the reason for the high emissions in both the wet and dry seasons, with biomass burning upwind of SAN representing only 15% of total CH_4_ flux in the dry season; anthropogenic emissions represent around 11% of the annual mean flux.

The 13 year time series of CH_4_ fluxes exhibits some interannual variability and revealed larger emissions between 2007 and 2013 than during 2000 and 2006. The largest emissions were in 2008, with emissions 3 Tg CH_4_ higher than in 2007, representing 19% of the global increase observed in that year. We highlight that SAN is located in the eastern part of Amazon Basin, and comparison to a state of the art estimate of emissions from the rest of the basin by *Melack et al.* [2004] suggests that emissions from the eastern Basin may be significantly larger than the rest of Amazon.

## Appendix A

Figure A1 shows the location of 14 surface stations from the INMET network located upwind of SAN used to calculate precipitation and temperature in this study.

The CH_4_ background mole fraction obtained for each profile is shown for SAN together with the two global monitoring sites ASC and RPB (Figure A2), indicating that the air entering to the continent receives more contribution from the air coming from the south than from the north and is following the global increase in atmospheric CH_4_.

Figure A3 shows two profiles measured during the dry season used to calculate the CO:CH_4_ emission ratio. During the beginning of wet season (January and February) we observed emissions from biomass burning in SAN. These emissions come from fires in the northeastern Brazilian coast (Figure A4).

Figure A5 shows a weak correlation between monthly mean biogenic flux and precipitation (*r* ^2^ = 0.06, *p* value of 0.0048), between January and December, with an anticorrelation with temperature (*r* ^2^ = 0.03, *p* value of 0.0900).

Figure A6 shows the threshold of ±0.5°C for the Oceanic Niño Index (ONI) that indicates the occurrence of the El Niño and La Niña events and the SAN CH_4_ flux time series.
